# Bridging Gaps in Perinatal Mental Health: A Review of Peer and Non-specialist Supports for Racially, Ethnically and Culturally Diverse Communities in the U.S.

**DOI:** 10.1007/s11920-026-01692-2

**Published:** 2026-06-26

**Authors:** Antonella Onofrietti Magrassi, Anahí Collado, Laurel M. Hicks, Samuel Hubley, Desiree Bauer, Sona Dimidjian

**Affiliations:** Renée Crown Wellness Institute- University of Colorado-Boulder, 1135 Broadway, Boulder, CO 80302 USA

**Keywords:** Perinatal mental health, Peer support, Non-specialist providers, Task sharing, Social support, Diverse populations

## Abstract

**Purpose of review:**

This narrative review synthesizes findings from U.S.-based peer-reviewed studies published between January 2020 and May 2025 on peer and non-specialist delivered perinatal mental health support programs for racially, ethnically and culturally diverse populations.

**Recent findings:**

Eight quantitative studies demonstrated improvements in depression, anxiety, and parenting outcomes through mechanisms including behavioral activation, cognitive behavior therapy, increased social support, and self-efficacy. Structured peer and non-specialist delivered programs, emphasized social support and culturally resonant approaches, with higher session “dose” linked to grater improvement in mental health outcomes. Trauma-informed models and multi-component interventions integrating culturally adapted cognitive behavior therapy and navigation also reported positive outcomes. Qualitative findings reinforced these mechanisms, with participants highlighting the importance of cultural resonance, trust, and emotional connection. However, no studies conducted formal mediation analyses.

**Summary:**

Peer‑ and non‑specialist‑delivered perinatal mental health supports show promise in improving outcomes among racially, ethnically, and culturally diverse populations in the U.S. These models draw on behavioral, psychosocial, and relational approaches, however more research is needed to establish effectiveness, guide dissemination, and identify mechanisms of change. Implementation science is critical for advancing scalable models of training and supervision, while policy innovation is needed to create sustainable funding and reimbursement pathways to embed these roles into routine perinatal care. Future efforts should intentionally employ participatory research approaches, ensure cultural relevance, and strengthen partnerships with community‑based organizations to support implementation and maximize equity, feasibility and acceptability.

## Introduction

Perinatal mental health disorders (PMHDs), including depression, anxiety, posttraumatic stress, and related conditions, are the most common complications of pregnancy and childbirth in the United States [1]. These disorders affect over 20% of women in the perinatal period, with higher rates observed in the postpartum period compared to during pregnancy [2–4]. PMHDs are associated with myriad negative consequences both for mothers and their offspring, including increased risk of impaired maternal functioning, disrupted parent–infant bonding, and long-term cognitive and emotional difficulties in children of depressed mothers [5, 6]. Racially, ethnically, and culturally diverse populations are at heightened risk for PMHDs [7, 8]. For example, prevalence rates of postpartum depression among Black and Latina mothers range from 35% to 67%, substantially higher than rates observed in White populations [9, 10]. These disparities are not simply attributable to individual risk factors but rather reflect the impact of broader structural inequities such as institutional racism, immigration-related stress, language barriers, and limited access to culturally and linguistically responsive care [11–13].

Despite the high prevalence and increased risk of adverse correlates and consequences of PMHDs, many pregnant and postpartum women, particularly those from racially, ethnically, and culturally diverse backgrounds are underserved at multiple points of the mental health care continuum—including screening, diagnosis, and treatment [14]. These populations are less likely to be accurately identified through standard screening tools, less likely to receive formal diagnoses, and less likely to access timely, effective, and culturally responsive treatment through traditional health systems [11, 15]. These disparities extend to psychological treatment; racially, ethnically and culturally diverse perinatal women are less likely to be referred to or engage in psychotherapy often due to linguistic and cultural barriers, limited social support, stigma, work and/or family constraints and distrust on formal mental health services [16]. Even when psychotherapy is available, many providers report insufficient training in culturally responsive care and both clients and providers may experience discomfort raising issues of race, culture and ethnicity during sessions [17]. These gaps highlight the need for alternative models such as peer- and non-specialist–delivered interventions that offer greater cultural alignment, accessibility, and flexibility.

Obstetric providers often report limited confidence, time, and training to address PMHDs, mental health services are commonly siloed from obstetric care [18, 19] and these barriers to care are even more pronounced in maternity care deserts serving vulnerable and diverse populations [20].

Social support is one of the most robust and modifiable protective factors for PMHDs [21, 22]. While social support benefits all, perinatal individuals from diverse backgrounds often face distinct challenges in accessing support due to disrupted social networks from migration, experiences of discrimination, and acculturation-related changes to traditional caregiving roles (e.g., loss of extended family postpartum care, shifting from multigenerational to nuclear family caregiving, earlier return to work etc.; [23]. At the same time, collectivist values and community-based networks that are valued in many cultures can serve as powerful sources of strength and resilience when effectively engaged but often are less available for diverse perinatal individuals as a result of relocation and acculturation [24, 25].

Task-sharing of mental health care, defined as the redistribution of tasks typically reserved for specialist providers to trained lay providers, has been increasingly a focus of attention as a promising strategy to increase access to social and mental health supports [26, 27]. Perinatal mental health task-sharing approaches commonly include two key provider groups: peers and non-specialists. Peers are distinguished by their lived experience with perinatal mental health concerns, and challenges navigating related structural stressors such as poverty, racism, migration, or trauma [28, 29]. They are often mothers who faced mental health challenges during pregnancy or postpartum, and are trained to offer emotional, informational, and practical support. Additionally, peer-delivered programs are distinguished by being grounded in shared lived experience and cultural alignment, and often are associated with high acceptability among participants [30, 31] These features can increase trust, reduce stigma, and improve engagement among populations historically underserved by traditional mental health services [32, 33] In contrast, non-specialist providers may not have such lived experience but often are already embedded into the target community, and are trained to deliver psychosocial interventions in an accessible, culturally responsive way [34]. Non-specialists can include community health workers (CHWs), doulas, promotoras and nurses.

While terminology, roles and training, may vary across programs, peers and non-specialists are unified by shared characteristics such as being non-licensed to provide mental health services, community-based, and employing a reciprocal relationship which emphasizes trust, respect and collaboration [35, 36] In addition to these characteristics, the flexibility of these approaches allows non-specialists and peers to adapt to varying contexts and settings, making them especially well-positioned to engage underserved or hard to reach populations [37].

Although global evidence supports the effectiveness and acceptability of peer and non-specialist delivered mental health support programs in improving perinatal mental health across diverse samples, research in the U.S. remains limited and studies centering diverse communities and culturally tailored models are scarce [34, 38–44]. Given the sociopolitical and structural factors shaping perinatal experiences in the U.S., research focused on peers and non-specialists is essential to inform the design, implementation, and scaling of equitable care that is accessible and effective.

## Scope and Review Approach

This review synthesized findings from U.S.-based, peer-reviewed studies published between January 2020 and May 2025 that examined peer and non-specialist delivered perinatal mental health support programs for diverse populations. It focused on empirical studies evaluating the effectiveness, implementation, or participant experiences of peer and non-specialist delivered perinatal mental health programs, defined as those occurring from pregnancy through five years postpartum. To be included, studies had to: (a) evaluate a peer or non-specialist delivered program; (b) be conducted in the U.S.; (c) include racially or ethnically diverse participants or report race/ethnicity as a moderator; (d) report outcomes related to mental health, parenting, or service engagement; and (e) be peer-reviewed, published in English between 2020 and 2025. We excluded studies that focused solely on general support or non-mental health outcomes, those involving only licensed or specialist level providers, and non-empirical publications.

Data extraction was completed by the first and fifth authors. Extracted data included publication details, study location and design, sample characteristics (e.g., percentage of racially or ethnically minoritized participants), mental health measures, peer support program characteristics, delivery format and setting, key findings, and implications for practice. A total of ten studies met inclusion criteria: six randomized controlled trials, two qualitative studies, one open trial, and one hybrid quasi-experimental study. Key characteristics and findings of peers and non-specialist delivered programs are summarized in Tables 1 and 2.

**Table 1:** Summary of studies employing peer providers to deliver perinatal mental health support programs (*N* = 3)

Author (year)	Study Design	Location	Sample Characteristics	PMH^1^ Measures	Program Components	Program Delivery	Provider Characteristics
Collado et al. 2025 [29]	OT^2^	Colorado	*N* = 126 Spanish-speaking Latina women in Colorado, pregnant, postpartum, or with a child < 5; PHQ-9 ≥ 5	PHQ-9; GAD-7; PSS-10; BADS-SF	Alma: peer mentoring program grounded in behavioral activation (BA) and co-designed via participatory methods; 6–8 weekly 1:1 sessions (plus 2 optional boosters)	Delivered in-person or virtually by Spanish-speaking Latina peers	Peers (*compañeras de apoyo*): Latina/Hispanic mothers residing in Colorado, Spanish speaker with history of perinatal depression.
Lutenbacher et al. 2023 [52]	RCT^3^	Tennessee	*N* = 132 pregnant Hispanic women ≤ 26 weeks gestation enrolled during pregnancy; Spanish-speaking participants included	EPDS, PSI-SF	Maternal Infant Health Outreach Worker (MIHOW): program offering monthly home visits and group education sessions from pregnancy through early childhood	Delivered in-person by peer home visitors trained through the MIHOW model	MIHOW home visitors: mothers from the target community (same culture and/or language group) have strong problem solving and communication skills, and completed all MIHOW training
McHale et al. 2023 [45]	RCT^3^	Southeastern US City	*N* = 138 low-income, unmarried mother and father dyads (70 intervention, 68 control); at least one parent had to identify as Black; 77% of mothers were Black, 19% Caucasian, 4% mixed-race	EPDS	Focused Co-Parenting Consultation (FCC): six interactive, psychoeducation and skill-based sessions to strengthen co-parenting competencies.	Delivered prenatally at community sites each parenting couple matched with a community mentoring team (one female mentor, one male mentor)	Black paraprofessionals who shared the lived experiences of participating parents served as the primary mentors in the intervention.

^1^ Perinatal Mental Health (PMH)

^2^ Open-Trial (OT)

^3^ Randomized Controlled Trial (RCT)

*BADS-SF* Behavioral Activation for Depression Scale – Short Form, *EPDS* Edinburgh Postnatal Depression Scale, GAD-7 Generalized Anxiety Disorder – 7-item scale, *PSI-SF* Parenting Stress Index – Short Form, *PHQ-9* Patient Health Questionnaire – 9-item scale, *PSS-10* Perceived Stress Scale– 10-item scale

**Table 2:** Summary of studies employing non-specialist providers in perinatal mental health support programs (*N* = 7)

Author (year)	Study Design	Location	Sample Characteristics	PMH^1^ Measures	Program Components	Program Delivery	Provider Characteristics
Mersky et al. 2022 [46]	RCT^2^	Wisconsin	*N* = 237, low-income pregnant women enrolled in home visiting programs	PSI, EPDS	Healthy Families America: Services included screening and assessment, family goal planning, parenting guidance, social support, and referrals to community-based resources. Intensity: 1-hour weekly visit up to 6 months postpartum at minimum.	In person, through home visiting programs	Typically led by family support workers with auxiliary support from nurses.
Quiray et al. 2024 [47]	Qualitative	Washington	*N* = 9 doulas participated in focus group (3 Latina/Hispanic, 2 White, 4 Black) and *N* = 9 clients participated on in depth interviews. All clients were postpartum at the time of the interview (1 White, 3 Black and 5 Latina/Hispanic interviewed in Spanish).	NA^*^	Doulas provided culturally congruent support including advocacy, translation, decision-making guidance, emotional support, and informal mental health assistance; approaches to PMH/SUD^3^ varied by training and lived experience.	Delivered in person by community-based doulas through established, trust-based relationships; support was tailored to client needs during pregnancy and postpartum, often extending beyond birth-related care.	Doulas providing perinatal services to underserved communities through non-profit organization.
Singla et al. 2022 [17]	Qualitative	Toronto, Ontario, Canada; North Carolina; and Illinois, USA	*N* = 23 perinatal women with EPDS ≥ 10, English or Spanish-speaking and *N* = 28 treatment providers (15 non-specialists, 13 specialists) from the SUMMIT trial.	NA^*^	BA: brief, evidence-based treatment delivered by specialists and non-specialists; supported by session manuals, training, and supervision; adapted for English and Spanish speakers; included skill-building (e.g., activity scheduling, problem solving) and weekly sessions offering social connection.	Delivered by both non-specialist (e.g., nurses, midwives) and specialist (e.g., social workers, psychologists, psychiatrists) providers across sites, using both in-person and telehealth formats.	Non-specialists (*n* = 15; nurses, midwives) with no formal mental health training, and specialists (*n* = 13; social workers, psychologists, psychiatrists).
Singla et al. 2025 [48]	RCT^2^	Toronto, Ontario, Canada; North Carolina; and Illinois, USA	*N* = 1,230 adult women (≥ 18 years) with EPDS ≥ 10; pregnant up to 36 weeks or 4–30 weeks postpartum; English or Spanish-speaking; mean age = 33.27; 47.15% BIPOC; 54.49% nulliparous; 85.73% with history of depression/anxiety; 23.49% on psychotropic medications at enrollment	EPDS, GAD-7	BA delivered by specialists and nonspecialists across four arms	BA delivered via telemedicine or in person; higher session attendance in telemedicine arms	Nonspecialists without formal mental health training; demonstrated noninferior outcomes compared to specialists across depressive and anxiety symptom measures
Sperlich et al. 2022 [49]	RCT^2^	Buffalo, NY	*N* = 56 pregnant women (ages 20–25) with low income, limited education, and trauma histories; majority identified as African American, with additional representation from Latinx, European American, Asian, multiracial, and other backgrounds	ACEs, EPDS, PCL-C, SCL-90-R, STAXI-AX, SUD	Survivor Moms’ Companion (SMC): a 10-module, trauma-informed, psychoeducational program for pregnant trauma survivors focused on coping skills, emotion regulation, and understanding PTSD^4^; delivered with weekly tutor support	Delivered in person by CHWs trained through the Buffalo Prenatal-Perinatal Network; CHWs met weekly with participants for 30–60-minute tutoring sessions	CHWs with shared cultural/linguistic backgrounds and standardized training; selected CHWs received specialized training in SMC and reflected the client population’s diversity
Tandon et al. 2021 [50]	RCT^2^	Iowa, Illinois, Michigan, Minnesota, Missouri, Ohio, and West Virginia	*N* = 824 (149 control, 293 MHP, 382 HVP); mean age = 26.3; 70% racial/ethnic minority; 13.3% received the intervention in Spanish; 11.77% foreign-born	BADS, MMS, NMRS QIDS-SR16	Mothers and Babies (MB) 1-on-1; a cognitive-behavioral, manualized intervention focused on mood regulation, cognitive restructuring, pleasant activities, and social support; 12 sessions of 15–20 min each	Delivered by either mental health professionals (MHPs) or home visitors (HVPs) trained in MB program	MHPs had ≥ 5 years of mental health experience; HVPs had no more than a bachelor’s degree in a related field; 24.5% Black/African American, 50.9% White, 22.6% Hispanic/Latina
Tandon et al. 2022 [51]	Quasi-experimental	Florida	*N* = 1,229 pregnant women (557 intervention, 672 control); ≤32 weeks gestation; ≥16 years old; mild-to-moderate depressive symptoms or identified as at-risk; English or Spanish speakers	BADS, BDI, EPDS, NMRS, PSS-4	Mothers and Babies (MB) 1-on-1; a cognitive-behavioral, manualized intervention focused on mood regulation, cognitive restructuring, pleasant activities, and social support; 12 sessions of 15–20 min each.	Delivered by lay home visitors during regular home visits; most sessions held weekly or bi-weekly over 3–6 months; train-the-trainer model used for implementation	Predominantly female home visitors with diverse racial/ethnic backgrounds (African American, Latina, White); many had > 3 years of experience; backgrounds include nursing, doula work, case management, and community health

^1^ Perinatal Mental Health (PMH)

^2^ Randomized Controlled Trial (RCT)

^3^ Substance Use Disorder (SUD)

^4^ Posttraumatic Stress Disorder (PTSD)

* Not applicable (NA)

*ACEs* Adverse Childhood Experiences Questionnaire; BA = Behavioral Activation, *BADS* Behavioral Activation Depression Scale, *BDI* Beck Depression Inventory-II, *EPDS* Edinburgh Postnatal Depression Scale, *GAD-7* Generalized Anxiety Disorder 7-item scale, *MMS * Maternal Mood Screener, *NMRS* Negative Mood Regulation Scale, *PHQ-9* Patient Health Questionnaire 9-item scale, *PSI* Parenting Stress Index, *PSS-4* Perceived Stress Scale – 4-item scale, *PCL-C* PTSD Checklist-Civilian; *QIDS-SR16* Quick Inventory of Depression Symptomatology-Self-Report, *SCL-90-R* Symptom Checklist-90-Revised, *STAXI-AX* State-Trait Anger Expression Inventory – Anger Expression subscale, *SUD* Subjective Units of Distress

## Current Evidence

We identified eight quantitative studies that met inclusion criteria. Studies were conducted exclusively in the United States, except for one with site in both U.S. and Canada. All included racially, ethnically and culturally diverse populations: four were conducted primarily among Spanish-speaking Latina participants, three centered on Black perinatal individuals, and one included a diverse sample with representation from multiple groups. We organized findings based on the type of program provider, distinguishing two groups: Peers (i.e., shared lived experience or cultural background with participants) and non-specialist providers (i.e., doulas, nurses, and community health workers).

### Quantitative Evidence Supporting Peer Delivered Programs

Three of the eight quantitative studies utilized peer mentors as providers. These peer-led programs drew on diverse therapeutic approaches, including behavioral activation (BA), psychoeducation, and hybrid parenting models. Delivery settings varied across studies and included home visits, community centers, and telehealth platforms.

*Alma*, a peer-delivered, BA program, delivered in 6–8 (plus two optional booster sessions) weekly one-on-one sessions, was associated with significant reductions in depressive symptoms, anxiety, and perceived stress among Spanish-speaking Latina mothers, with the most notable improvements occurring during the first three sessions [29]. In a randomized clinical trial targeting pregnant Latinas, peer mentors delivered the Maternal Infant Health Outreach Worker (*MIHOW*) program which consisted of monthly home visits and educational groups from pregnancy through early childhood. *MIHOW* demonstrated sustained and significantly greater improvements over time in depressive symptoms, maternal stress, emotional support, and parenting self-efficacy compared to controls [52]. Another study investigated the Focused Co-parenting Consultation (*FCC*) program, a strengths-based, psychoeducational co-parenting program delivered by trained Black paraprofessional community mentors to low-income mother-father dyads. Reported outcomes showed reductions in depressive symptoms in both intervention and control groups, with only marginal evidence of greater improvement among those receiving the peer mentor intervention [45].

### Quantitative Evidence Supporting Non-Specialist Delivered Programs

Five studies examined programs delivered by non-specialist providers, including community health workers, doulas, nurses, and paraprofessional family support workers. These programs incorporated diverse therapeutic modalities including BA, cognitive behavioral therapy (CBT), and trauma-focused psychoeducation. Delivery settings ranged from home-visiting programs to telehealth platforms and real-world service environments.

Three studies demonstrated positive mental health outcomes. The *SUMMIT* trial evaluated a BA program delivered by non-specialists showed that non-specialist delivery was non-inferior to specialist clinicians; participants receiving telehealth completed more sessions and experienced significant reductions in depression and anxiety [48].The Survivor Moms’ Companion (*SMC*) program, provided trauma-focused modules through trained non-specialists, resulted in significant reductions in PTSD and depression symptoms, with greater benefits among participants who completed more modules [49]. Additionally, Mothers and Babies (*MB*) a home-visiting program targeting perinatal women at risk for poor birth outcomes, consisting of six weekly CBT-based sessions, yielded reductions in depressive symptoms and perceived stress compared to controls [51].

Two studies reported mixed or inconclusive findings. One study evaluated a home-visiting program delivered by paraprofessional family support workers and found variable effects on parental distress and depressive symptoms, with greater benefits observed among participants with higher baseline depression scores [46]. Another compared depressive symptom reduction among three groups: women receiving usual community-based services, women receiving *MB* from mental health professionals, and women receiving *MB* from paraprofessional home visitors. While *MB* did not significantly outperform usual care, the program delivered by home visitors was similarly effective to that delivered by mental health professionals. These findings suggest that trained paraprofessionals may be a viable option for delivering preventive mental health programs in community settings [50].

### Qualitative Insights on Implementation and Engagement

Several studies included qualitative data that enriched understanding of implementation and contextual factors influencing delivery. Participants and providers emphasized the importance of consistent emotional connection and trust, which facilitated engagement, especially during COVID-19 [53]. Doulas valued their roles not only in birth support but also in emotional validation, advocacy, and care navigation [47]. However, shared community ties sometimes complicated discussions about perinatal mental health and substance use due to stigma, causing hesitancy among providers to address sensitive topics [47]. Across studies, limited mental health training and unclear role boundaries emerged as significant barriers to optimal support in peer and non-specialist models [50, 53].

Further, the transition to telehealth during COVID-19 was identified as a potential facilitator to care. Telemedicine increased access and flexibility, particularly for participants facing childcare or transportation barriers, and peer mentoring programs successfully adapted to phone and video delivery while maintaining mental health benefits [29, 53].

Across studies, program delivery approaches shaped the extent to which logistical barriers were addressed. Home-based models including *MIHOW*,* SMC*,* MB* and the state-supported home visiting programs described by Mersky, reduced the need for transportation and enabled flexible scheduling [46, 49–52]. Programs that began in person, such as *Alma* and *FCC* shifted to virtual delivery during the COVID-19 pandemic, which further alleviated access constraints [29, 45]. Finally, findings from the *SUMMIT* trial reinforce that telemedicine can meaningfully reduce logistical barriers, particularly childcare and transportation, and is often preferred over in-person modalities among perinatal patients [48, 53].

### Mechanisms of Change

Across the eight quantitative studies, symptom improvement was associated with behavioral, psychosocial, and relational factors, although no studies conducted formal mediation analyses. Several trials explicitly monitored activity level, a putative mechanism of BA, and found it tracked closely with mood change. In the *Alma* open trial, every one‑hour peer‑mentoring session was associated with an average 4.7‑point gain on the BADS‑SF, and these gains paralleled declines in PHQ‑9 scores during the first six meetings [29]. Likewise, in the hybrid *MB* study, higher session “dose” predicted larger improvements in BA, coping expectancies, and perceived stress [51]. BA was also central to the *SUMMIT* non‑inferiority RCT, where completion of a mean six sessions, particularly in the telehealth arm, co‑occurred with significant reductions in depression and anxiety [48].

Several studies highlighted social support and self‑efficacy as plausible pathways. For instance, *MIHOW* participants reported progressively larger gains in perceived emotional support and parenting self‑efficacy, with effect sizes for both constructs growing from small at two months to large at 15 months postpartum; these psychosocial improvements mirrored sustained reductions in EPDS scores [52]. A study looking at the impact of a visiting home program on parenting stress showed that higher social support at baseline predicted lower parental distress across all arms, underscoring its protective role [46]. An RCT looking at *FCC* a prenatal, co‑parenting consultation program, tested targeted dyadic cooperation; qualitative feedback suggested that enhanced co‑parenting confidence, rather than educational content alone, helped mothers and fathers manage stress and depressive feelings [45].

Two trials focused on trauma and stress regulation. *SMC* was associated with shifts in perinatal PTSD and depression from clinical to non‑clinical ranges among women completing ≥ 40% of modules, providing suggestive evidence that structured psychoeducation and emotion‑regulation practice may be active ingredients [49].

Qualitative data was in line with these quantitative indicators. Weekly contact in BA sessions created consistent social connection that participants described as “a lifeline” during pandemic isolation (53). Doulas in Quiray and colleagues, and collaborators and peer mentors in *Alma* underscored how cultural and linguistic concordance fostered trust, normalized help‑seeking and lowered stigma, relationship qualities participants themselves linked to their willingness to practice new skills [29, 47]. At the same time, participants noted that these same shared community ties could reinforce existing stigma around mental health, sometimes making open discussions more difficult. This dual effect underscores the importance of structured training and supervision to support both culturally resonant and psychologically safe interactions [47, 50].

## Challenges and Promising Solutions of Peer and Non-Specialist Delivered Perinatal Mental Health Programs

Despite promising evidence that peer and non-specialist delivered approaches can address perinatal mental health gaps, important challenges exist and must be addressed for these to fully realize their potential of increasing access. Addressing barriers related to healthcare systems integration, community partnerships, workforce policies, training and supervision, cultural relevance, and participatory engagement in program design and refinement will be critical [26, 28, 44, 54]. This section outlines key challenges and strategies across five core implementation domains.

### Integration into Healthcare Systems and Medical Settings

Peer-led and non-specialist-delivered programs are often conceptualized along a continuum of mental health support. They can serve as prevention, early intervention, or adjunctive care for PMHDs, and in some cases even as stand-alone support when specialized care is unavailable, particularly among mothers facing structural barriers including insurance/cost, lack of culturally and linguistically competent providers, and lack of coordianted referrals [34, 55].

Integrating peer and non-specialist roles into primary care and healthcare settings has been demonstrated to be effective, feasible and acceptable as a strategy to respond to the increasing need for mental health care services [54, 56]. Within PMHD care, peer and non-specialist roles may complement existing services by enhancing cultural responsiveness, empowerment, trust, and stigma reduction—particularly in underserved communities for whom early identification and access to care is complex and full of systemic barriers [38, 57]. Research shows that in the U.S., screening for PMHDs in diverse populations is inconsistent, yet when screening is combined with interventions that address patient, provider, and system-level barriers, rates of detection, assessment, referral, and treatment improve [14, 58]. Non-specialists and peers may assist with screening, care coordination, psychoeducation, and advocacy; however, effective integration requires clear role definitions, interdisciplinary collaboration, and inclusive onboarding that values lived experience [59].

Several of the programs included in this review illustrate effective integration of peer and non-specialist roles within healthcare systems and medical settings. *SUMMIT* embedded BA into routine prenatal care delivered by nurses and midwives, and leveraged telehealth to increase reach [34, 48]. *MB* was incorporated into the state agencies protocols for home visiting programs, ensuring systematic screening and referral for subthreshold depression [50, 51]. Similarly, *Alma* established a strategic partnership with Colorado PROSPER, the state’s perinatal Psychiatric Access Program to build referral pathways from a variety of perinatal care providers to trained peer mentors through psychiatric consultation. This partnership plays a critical role in advancing equity, as Access Programs have demonstrated potential to improve perinatal mental health outcomes across multiple levels—including the patient, provider, practice, community, population, and policy levels—through collaboration with key community stakeholders [60].

### Establishing Partnerships with Community-Based Organizations (CBOs)

Many programs addressed the challenge of implementation in under-resourced community-based settings by establishing ongoing partnerships with the communities they intend to engage. *Alma* collaborated with three CBOs—two urban and one rural—and was co-designed with perinatal people, community leaders and medical providers to ensure fit and feasibility [29]. Currently, the *Alma* team conducts an organizational assessment with potential CBO partners to ensure alignment with program values, structural capacity to support peer roles, and leadership commitment to implementation. This step informs training logistics and supervision planning and helps identify potential barriers to sustainability.

The *MIHOW* program relied on grassroots outreach, with bilingual staff recruiting through local agencies and businesses, supported by ‘site leaders’ in each locality who oversees recruitment, training, supervision, fundraising and community representation [52, 61].

The *SUMMIT* research team established strong partnerships with diverse stakeholders across North America, including individuals with lived experience, community partners, clinicians, insurers, and policymakers. Engagement strategies included forming a Stakeholder Advisory Committee and having an ongoing process of evaluation to ensure content and materials are acceptable and feasible from multiple perspectives. These partnerships also guide inclusive dissemination through press releases, local presentations, digital materials, policy briefs, and academic and non‑academic publications [62].

*MB*, in the context of home visiting programs, was developed through strong academic–community partnerships in which researchers worked closely with community leaders and stakeholders, incorporating extensive feedback from perinatal patients, home visiting staff, and a community advisory board [50].

Lastly, *FCC* and *SMC* were delivered by trusted leaders and community health workers (CHWs) embedded in established local organizations, reflecting the importance of social capital and community trust for uptake and sustainability [45, 49].

### Workforce Development

Sustainability of peer and non-specialist delivered supports in the perinatal context depend on secure reimbursement mechanisms, equitable compensation, and formal credentialing pathways [54]. In the U.S., Medicaid reimbursement for peer services is available in over 40 states, but coverage of perinatal-specific peers remains inconsistent [63] and the reimbursement rates are lower than necessary for a livable wage. There is a growing push to create perinatal peer certification programs and integrate peer services into value-based payment models. A recent report by the SAMHSA underscores the importance of developing national standards for peer credentialing that are inclusive of diverse pathways into the workforce [64]. However, language remains a structural barrier for many non-English-speaking peers, as credentialing exams are typically only available in English. Without linguistically accessible certification options, these peers may be excluded from formal recognition and career advancement opportunities. Countries such as Canada and the UK have begun incorporating peer and non-specialist roles into national maternal mental health strategies [40, 65]. Ensuring that non-specialists and peers are compensated fairly, offered career advancement opportunities, and protected from precarity as this is vital for workforce development and program efficacy.

To address shortages of culturally responsive perinatal mental health providers, all reviewed programs recruited and trained individuals from the target communities. *Alma* trained both Spanish and English-speaking community members with lived experience, *MIHOW* and *SMC* leveraged CHWs who matched participants linguistically and culturally, and *FCC* prioritized Black paraprofessionals with community ties, high social capital (e.g. elders, pastoral counselors) and shared parenting experiences [29, 45, 49, 52]. *MB* and *SUMMIT* created career progression pathways to create mobility from implementer to trainer, expanding workforce capacity and enabling long-term scalability [48, 50, 51].

### Training, Supervision, and Certification

High-quality training and supervision emerged as an essential component of program fidelity and effectiveness. Several of the reviewed programs, peers and non-specialists operated within clearly defined safety and referral protocols that interfaced directly with licensed clinicians when elevated concerns emerged. In *Alma*, any endorsement of suicidality triggered immediate follow-up by a licensed mental health provider, who conducted risk assessment and facilitated referrals to crisis services and the participant’s healthcare provider [29]. In *MIHOW*, data collectors were trained to flag high EPDS scores, suicidal ideation, and child abuse indicators and to notify the program coordinator who initiated referrals to mental health or child-protection services [52]. Mersky’s home-visiting program relied on a dyadic model pairing a paraprofessional and a public health nurse explicitly recognizing that home visitors serve families with complex needs and must link them to external clinical and community services through structured screening and referral pathways [46]. Tandon and colleagues describe the development of a standardized crisis protocol co-designed with stakeholders, outlining procedures for *MB* home visitors when participants disclosed safety concerns and specifying when issues must be escalated to the research team or local clinical partners [50]. In *SMC* non-specialists frequently connected clients to healthcare providers or community supports, although the study did not track referrals systematically [49]. The *SUMMIT* trial embedded non-specialist delivery directly within obstetric and psychiatric care pathways, with referrals originating from clinical teams and safety overseen by site psychiatrists, principal investigators, and the study’s Data Safety and Monitoring Board (DSMB); non-specialists received regular supervision and were required to alert clinical leads about any potential adverse events [48].

Across these models, peers and non-specialists were not expected to diagnose or manage high-acuity mental health needs, and these concerns were escalated to clinicians via predetermined protocols and referral pathways. Yet, the studies reviewed also reveal gaps; referral responsibilities varied widely across programs, documentation of referral follow-through was inconsistent, and several programs did not fully specify how peers/non-specialists and clinicians communicated beyond crisis escalation. More explicit delineation of collaborative workflows, information-sharing processes, and ongoing supervisory structures is needed to ensure integration of peer roles within the broader perinatal mental health care system.

Programs like *Alma* and *MB* used train-the-trainer models to promote sustainability. *Alma* emphasized experiential learning, ongoing reflective supervision, and certification based on fidelity and competency [29]. In the *Alma* program, training begins with peer mentors completing the *Alma* program as participants to reflect on their own experiences. They then engage in 76 h of structured training, which includes content on BA, confidentiality limits, validation, asking open-ended questions, nonjudgmental engagement, agenda adherence, and reflections. Training also incorporates a minimum of five “mock mom” sessions, during which peer mentors practice facilitating sessions and receive feedback from the *Alma* team, including licensed mental health professionals and experienced peer mentors. Certification is based on demonstrated fidelity, competency, and readiness, assessed using standardized evaluation forms. Ongoing support includes weekly reflective supervision sessions focused on program fidelity, peer well-being, and continuous skill development. Refresher consultations, especially regarding risk assessment and safety protocols, are provided regularly, along with individualized support when needed. This multi-layered infrastructure, co-developed with community input, has contributed to high rates of fidelity and organizational commitment to implementing *Alma*.

The *MB* program trained facilitators (home visitors) using a standardized process that aims to balance fidelity and delivery flexibility. *MB* facilitators received a one- to one-and-a-half-day training from the Principal Investigator (PI) delivered either in-person, live webinar, or pre-recorded webinar with additional follow-up calls [50, 51]. The program prepares *MB* facilitators for one-on-one and group delivery with some receiving additional phone-based training to support modality transitions. This strategy has been linked to higher adherence compared to recorded training alone. All facilitators implementing their first cohort receive direct supervision from the PI [50].

*MIHOW* and *SMC* offered extensive initial training and ongoing support for CHWs, with structured supervision and fidelity checklists embedded in practice [45, 52]. These efforts ensure that peers and non-specialists were able to deliver programs with both sensitivity and accuracy. In *MIHOW*, outreach workers are women from the local community who are also mothers and complete at least 40 h of initial training in the model, after which they shadowed an experienced outreach worker before delivering services [52]. Training covers pregnancy, childbirth, infant feeding, child development, and positive parenting, along with *MIHOW* practices (e.g., listening to maternal concerns and tailoring education to the mother’s stage of pregnancy or child’s age) [66]. Ongoing training is required, and outreach workers must demonstrate a specific level of proficiency before conducting visits. Agencies implementing MIHOW must achieve *Commitment to Excellence MIHOW Accreditation Program* (CEMAP) status within three years, and renew every five years [67].

In *SMC*, tutors completed a structured three-day training covering the program’s foundational research, the effects of trauma, principles of trauma-informed care, and the program materials. Upon completion, tutors were fully prepared to deliver the program. Project-specific training was also provided to agency staff and supervisors to ensure alignment and support. Ongoing supervision was a core component of the model, with agency social workers providing regular guidance and oversight to maintain fidelity and support tutors in navigating trauma-related content [49].

Finally, the *SUMMIT* team provided structured training to both non‑specialist and specialist providers to deliver BA for perinatal depression and anxiety. For non‑specialist providers, a four‑day training began with an introduction to the trial, background on perinatal depression and anxiety, administration of mood measures (EPDS, GAD‑7), and foundational therapy skills. Subsequent training covered BA sessions, safety protocols, and concluded with a multiple‑choice exam, competency role‑play, and training evaluation. Specialist providers received equivalent training with expanded BA coverage. This parallel but tailored approach ensured both groups met competency requirements while accommodating differing backgrounds and experience [62]. Future research will benefit from greater detail regarding program training, supervision, and certification practices.

### Cultural Relevance

For many collectivistic cultures, postpartum care traditionally relies on multigenerational and communal caregiving structures, such as extended family and community that provide emotional and practical support [68]. These culturally rooted practices typically help reduce maternal stress and foster well-being; however, migration, consequent separation from family and support systems, and acculturation can disrupt, challenge, or erode these traditions, increasing isolation and stress for mothers in the perinatal period [69].

Peer approaches may address this need as peer relationships offer social connection with someone who shares lived experience—a known protective factor against depression, anxiety, and maternal stress [70]. Moreover, peers function in a “liminal” role where they are neither a client nor a provider but have lived experience and formal training that enables culturally resonant engagement, especially in communities facing medical mistrust and systemic barriers. Culturally matched peers (e.g., promotoras, compañeras) can normalize mental health struggles and reduce stigma [31].

Given the importance of cultural relevance, most programs incorporated adaptations as core components. *Alma*’s co-design approach centered the voice of Latina mothers through focus groups and collaborative design activities that invited them to share preferences for perinatal care and guide program design [27]. Similarly, a qualitative study showed that *Alma* peers *(compañeras)* draw from their cultural knowledge to build trusting relationships rooted in the cultural value of *confianza*, create safe spaces for healing and personalize *Alma* in a way that is culturally relevant [33]. *MIHOW*, *SMC* and *MB* recognized the importance of cultural relevance and emphasized cultural matching between participants and lay providers, although these programs did not report adapting their core content [49–52]. *FCC* was co-created with Black families and community leaders and delivered in trusted community settings [45]. Overall, these culturally grounded practices increased engagement, normalized help-seeking, and promoted mental health equity.

### Participatory Approaches to Design and Refinement

Participatory approaches, such as co‑design, co‑creation, and co‑production methods, are increasingly recognized as promising strategies in health research, particularly when working with diverse populations [71, 72].These approaches aim to bridge gaps between research priorities and patient need, ensuring that the work is not only evidence-based but also community-informed and aligned with the specific needs and strengths of diverse populations.

Given inconsistent definitions of participatory approaches, we use the model proposed by Grindell et al. to guide the following section [73]. This model outlines key aspects of participatory work: bringing people together as active, equal partners; valuing diverse forms of knowledge; using creative methods; and engaging in iterative processes. These key aspects activate mechanisms such as shared understanding, meeting stakeholder needs, amplifying voice and power, fostering ownership, and building trust. In turn, these mechanisms produce more relevant, acceptable, and usable research products, enhance implementation in practice, and ultimately improve health outcomes [71]. This section summarizes how participatory approaches informed program design in some of the reviewed studies, and highlights their relevance to future efforts when designing peer and non-specialist models for diverse communities of perinatal women, grounded in equity, community engagement, and systems change [74–76].

*Alma* was co-designed through sustained engagement with community leaders, potential peer mentors, and mothers with lived experience of perinatal depression. Initial conversations in rural Colorado communities raised deep concern about increasing rates of depression and suicide among Latina mothers. In response, the *Alma* team conducted a series of focus groups that identified peer-delivered support as a promising strategy, particularly when paired with structured, evidence-based models [27]. These early insights sparked a collaborative co-design process, bringing together researchers, community members, and women with lived experience to shape the program content, delivery model, and training approach. The program was then piloted and iteratively refined across both medical and community settings, with feedback loops guiding each stage of development. By embedding co-design into all levels of development and delivery, the *Alma* program offers on example of the potential of partnering with communities not only to deliver mental health support, but to reshape how behavioral health care is designed, experienced, and sustained.

*MB* incorporated participatory research aspects by embedding patients and stakeholders in decision‑making throughout the project. The team created three distinct advisory boards to obtain ongoing input. The first was an operations team, which involved a government official, home visiting program managers, staff and clients, two *MB* group facilitators (one peer, one mental health provider) and one patient advocate; this group met quarterly to guide design, implementation, and dissemination. The second group was an executive committee of key stakeholders that met bi‑weekly with investigators to ensure timely achievement of milestones. The third group was a national advisory committee of experts in postpartum depression, home visiting and policy, and patient advocates that met annually to identify opportunities for broader dissemination and sustainability. These multi‑level, sustained collaborations ensured the research was shaped by diverse perspectives and aligned with community and stakeholder priorities [50].

Finally, *SMC* was developed by midwives in partnership with survivor mothers from diverse backgrounds using participatory action research (PAR) to ensure the program reflected survivors lived experiences and priorities. This process valued diverse knowledge through direct inquiry. Survivors identified key needs such as avoiding medical triggers, fostering trust with maternity providers, addressing parenting challenges in the context of limited support, and integrating care. The main goal of this approach was to ensure that the final model was relevant, acceptable, and feasible [77].

The use of participatory approaches in perinatal mental health programs show promise for enhancing relevance, acceptability, and sustainability. However, their application varies widely, underscoring the need for clearer definitions, consistent reporting, and intentional integration in developing and evaluating peer and non-specialist supports for PMHDs.

## Conclusion

The reviewed studies demonstrate the promise and strong potential of task sharing models to reduce PMHDs in diverse populations by training peers and non-specialists to deliver perinatal mental health support and care in the U.S. These programs leveraged from well documented evidence-based theories and models, such as psychoeducation, BA and CBT, and paired that with the relational power of peer and community-based providers to enhance engagement and effectiveness. The extant evidence base provides a strong foundation from which the field can advance research designs and methods to test the effectiveness of peer and non-specialist provider programs across diverse populations. Moreover, there is a need for additional research to examine mechanisms of change. To extend these promising models into equitable, sustainable care, future efforts also must address structural and systemic challenges related to healthcare integration and community partnerships, as well as workforce development, training and supervision pathways. These efforts will be strengthened by prioritizing cultural relevance and participatory approaches to program design and refinement. The studies in this review provide instructive and hopeful models from which future work should build. Expanding research and strengthening infrastructure for peer and non-specialist provider mental health support is essential to advancing perinatal mental health equity across diverse U.S. populations.

## Key References

- Hall SV, Zivin K, Piatt GA, Weaver A, Tilea A, Zhang X, et al. Racial Disparities in Diagnosis of Postpartum Mood and Anxiety Disorders Among Symptomatic Medicaid Enrollees, 2012–2015. Psychiatr Serv. 2024 Feb 1;75(2):115–23.
  - ○ This study provides insight into the detection gap in postpartum mood and anxiety disorders that are formally diagnosed among symptomatic Medicaid enrollees in Michigan.
- Kozhimannil KB, Trinacty CM, Busch AB, Huskamp HA, Adams AS. Racial and Ethnic Disparities in Postpartum Depression Care Among Low-Income Women. Psychiatr Serv. 2011 Jun 1;62(6):619–25.
  - ○ This study highlights disparities in postpartum depression care by examining mental health service use among a multiethnic cohort of Medicaid recipients.
- Matthews K, Morgan I, Davis K, Estriplet T, Perez S, Crear-Perry JA. Pathways To Equitable and Antiracist Maternal Mental Health Care: Insights from Black Women Stakeholders: Study examines pathways to equitable and antiracist maternal mental health care. Health Aff (Millwood). 2021 Oct 1;40(10):1597–604.
  - ○ This qualitative study identifies five critical pathways to advance racial equity in maternal mental health, emphasizing investment in Black women practitioners, community organizations, and culturally rooted care models.
- Alsamman S, Tadesse RT, Sarferaz K, Mohamed AS, Mody SK. Barriers to postpartum health and opinions on a postpartum peer navigator program amongst refugee women resettled in California. BMC Pregnancy Childbirth [Internet]. 2025 Mar 29 [cited 2025 Jul 21];25(1). Available from: https://bmcpregnancychildbirth.biomedcentral.com/articles/10.1186/s12884-025-07479-2.
  - ○ This study explores postpartum needs among refugee women in California, highlighting interest in language-concordant peer navigation to address barriers in contraception and mental health care.
- Patel V. Scale up task-sharing of psychological therapies. The Lancet. 2022 Jan;399(10322):343–5.
  - ○ This commentary describes the global relevance of task-sharing models for psychosocial interventions, noting their growing adoption in high-income settings and emphasizing training and quality assurance as key implementation challenges.sds
- Watson E. The mechanisms underpinning peer support: a literature review. J Ment Health. 2019 Nov 2;28(6):677–88.
  - ○ This scoping review examines the processes and mechanisms of peer support, providing insight into how and why peer support works and offering guidance for optimizing its impact.
- Sangraula M, Chauhan J, Best C, McEneaney C, Shah C, Brown AD, et al. The impact of task-sharing scalable mental health interventions on non-specialist providers: a scoping review. Camb Prisms Glob Ment Health [Internet]. 2024 [cited 2025 Jul 22];11. Available from: https://www.cambridge.org/core/product/identifier/S2054425124001298/type/journal_article.
  - ○ This review maps the effects of task-sharing mental health interventions on non-specialist providers identifying both positive impacts and key recommendations.
- Liblub S, Pringle K, McLaughlin K, Cummins A. Peer support and mobile health for perinatal mental health: A scoping review. Birth. 2024 Sep;51(3):484–96.
  - ○ This scoping review synthesizes evidence on the use of mHealth and peer support for perinatal mental health.
- Shah L, Chua JYX, Goh YS, Chee CYI, Chong SC, Mathews J, et al. Effectiveness of peer support interventions in improving mothers’ psychosocial well‐being during the perinatal period: A systematic review and meta‐analysis. Worldviews Evid Based Nurs. 2024 Dec;21(6):652–64.
  - ○ This review provides evidence that peer support interventions during the perinatal period can significantly improve mothers’ psychological well-being and family health, supporting their integration as a standard component of perinatal care.
- Atif N, Bibi A, Nisar A, Zulfiqar S, Ahmed I, LeMasters K, et al. Delivering maternal mental health through peer volunteers: a 5-year report. Int J Ment Health Syst. 2019 Dec;13(1):62.
  - ○ This mixed-methods evaluation of a peer-delivered perinatal mental health program in rural Pakistan found sustained engagement and competency among 70% of peer volunteers five years post-implementation.
- Huang R, Yan C, Tian Y, Lei B, Yang D, Liu D, et al. Effectiveness of peer support intervention on perinatal depression: A systematic review and meta-analysis. J Affect Disord. 2020 Nov;276:788–96.
  - ○ This systematic review and meta-analysis found that peer support interventions show promise in preventing or reducing perinatal depression.
- Mendenhall E, Silva MJD, Hanlon C, Petersen I, Shidhaye R, Jordans M, et al. Acceptability and feasibility of using non-specialist health workers to deliver mental health care: Stakeholder perceptions from the PRIME district sites in Ethiopia, India, Nepal, South Africa, and Uganda. Soc Sci Med. 2014;118:33–42.
  - ○ This multi-country qualitative study found that task-sharing in mental health care is considered acceptable and feasible if key system-level supports are in place.
- Grindell C, Coates E, Croot L, O’Cathain A. The use of co-production, co-design and co-creation to mobilise knowledge in the management of health conditions: a systematic review. BMC Health Serv Res. 2022 Jul 7;22(1):877.
  - ○ This systematic review analyzes the rationale, mechanisms, and activities underlying ‘co-approaches’ in health research, and provide an overview of themes.

## Data Availability

No datasets were generated or analysed during the current study.
